# Invasive molecular prenatal diagnosis of alpha and beta thalassemia among Hakka pregnant women

**DOI:** 10.1097/MD.0000000000013557

**Published:** 2018-12-28

**Authors:** Heming Wu, Huaxian Wang, Liubing Lan, Mei Zeng, Wei Guo, Zhiyuan Zheng, Huichao Zhu, Jie Wu, Pingsen Zhao

**Affiliations:** a Clinical Core Laboratory; b Center for Precision Medicine, Meizhou People's Hospital (Huangtang Hospital), Meizhou Academy of Medical Sciences, Meizhou Hospital Affiliated to Sun Yat-sen University; c Guangdong Provincial Engineering and Technology Research Center for Molecular Diagnostics of Cardiovascular Diseases; d Meizhou Municipal Engineering and Technology Research Center for Molecular Diagnostics of Cardiovascular Diseases; e Meizhou Municipal Engineering and Technology Research Center for Molecular Diagnostics of Major Genetic Disorders; f Prenatal Diagnosis Center, Meizhou People's Hospital, Huangtang Hospital; g Guangdong Provincial Key Laboratory of Precision Medicine and Clinical Translational Research of Hakka Population, Meizhou, PR China.

**Keywords:** Chinese, genetic mutations, Hakka pregnant women, prenatal diagnosis, thalassemia

## Abstract

This study is a retrospective analysis of the prenatal genetic diagnosis results of fetuses with high risk of major thalassemia to provide information for clinical genetic counseling and to better control the birth of major thalassemia child in Hakka population. Totally, 467 fetuses in at-risk pregnancies were collected from Meizhou people's hospital from January 2014 to December 2017. Genomic DNAs were extracted from peripheral blood of the couples and villus, amniotic fluid or cord blood of the fetuses. DNA-based diagnosis was performed using polymerase chain reaction (PCR) and flow-through hybridization technique. Follow-up visits were done half a year after the fetuses were born. Around 467 fetus at-risk pregnancies were performed prenatal diagnosis. We detected 88 CVS samples, 375 amniocentesis fluid samples and, 4 cord blood samples. The 356 fetuses in α-thalassemia families consisted of 69 (19.38%) with Bart's hydrops syndrome, 20 (5.62%) fetuses with Hb H disease, and 184 (51.68%) fetuses with heterozygote. And the 111 fetuses in β-thalassemia families consisted of 31 (27.93%) thalassemia major, 51 (45.95%) fetuses with heterozygote. There are 13 fetuses with α+β-thalassemia, including 2 cases with severe β-thalassemia. DNA-based testing prenatal diagnosis of thalassemia was found to be highly reliable. Our findings provide key information for clinical genetic counseling of prenatal diagnosis for major thalassemia in Hakka pregnant women. Our work plays an important role in the prevention and control of thalassemia in Hakka population. We will also combine other techniques to further improve our molecular prenatal diagnostic capabilities, including the next-generation sequencing (NGS), Sanger sequencing and MLPA.

## 1 Introduction

Thalassemia is considered to be one of the most common genetic diseases in the world, with a high frequency in tropical and subtropical regions such as Mediterranean countries, Indian subcontinent, Middle East, North Africa, and South Ocean.^[1,2]^ There are high incidences in Guangxi, Guangdong, and Hainan province of China.^[3–6]^ Thalassemia is an autosomal recessive disease characterized by microcytic hypochromic anemia resulting from the congenital defect of human globin gene, resulting in inadequate or complete loss of the corresponding globin chain synthesis and the imbalance of the ratio between alpha chain and nonalpha chain in hemoglobin, giving rise to moderate or severe hemolytic anemia. Thalassemia is classified into 2 major types, including α- and β-thalassemia.^[7,8]^

Alpha-thalassemia is a hereditary hematological disorder caused by the deletion or dysfunction of alpha-globin genes in chromosome 16 contributing to the absence or inadequate synthesis of alpha-globin peptide chains.^[9,10]^ The normal genotype can be represented as αα/αα. Alpha-thalassemia is characterized caused by deletion or nondeletional mutation of one (-α/αα, α^T^α/αα or αα^T^/αα) or both (--/αα or -α/-α) α-globin genes. Severe alpha-thalassemia can cause fetal death at the time of birth, represented as Hb Bart's (γ_4_) disease (--/--). People with loss or inactivation of 3 α-globin genes (--/-α or --/α^T^α or --/αα^T^) which in adult life excess β globin also form active tetramer with the structure β_4_ (HbH). HbH disease is highly heterogeneous and there are many different rules about their pathogenesis.^[11–13]^

Beta-thalassemia is a group of hereditary blood disorder characterized by point mutations or, more rarely, deletions in the beta globin gene on chromosome 11, leading to reduction (β^+^) or absence (β^0^) in beta chains of hemoglobin synthesis, resulting in variable phenotypes ranging from severe anemia to clinically asymptomatic level.^[14,15]^

Thalassemia can be clinically divided into 3 forms according to severity: thalassemia major, indicating severe anemia and being transfusion-dependent; thalassemia trait, usually referring to those clinically asymptomatic carrier state; and thalassemia intermedia, to describe patients with a phenotype ranging in severity from severe anemia with hepatosplenomegaly and thalassemia-like bone modifications to moderate microcytic hypochromic anemia.^[16–18]^

Diagnosis of thalassemia is based on hematologic and molecular genetic detection. The therapeutic methods of thalassemia major include regular red blood cell transfusions, iron chelation and management of complications caused by iron overload. In some circumstances, spleen removal may be required. Bone marrow transplantation remains the only effectively curable measure currently available. Individuals with thalassemia intermedia may require splenectomy, folic acid supplementation, treatment of extramedullary erythropoietic masses and leg ulcers, prevention and therapy of thromboembolic events.^[19,20]^

Couples with similar thalassemia genes may more likely give birth to children with thalassemia major. Prenatal diagnosis can prevent the birth of children with thalassemia major. Prenatal diagnosis of thalassemia by molecular biology technology has been widely applied. Prenatal diagnosis is of great significance for thalassemia prevention. In Guangdong province, the prevalence of thalassemia is very high. Meizhou city is located in the eastern part of Guangdong province, in which the prevalence is higher than the average lever in Guangdong. The Hakka people are an intriguing Han Chinese populations that mainly inhabit southern China, including Meizhou city. In order to better prevent thalassemia in this area, we retrospectively analyzed the data of molecular prenatal diagnosis of alpha and beta thalassemia among Hakka population from 2014 to 2017.

## 2 Materials and methods

### 2.1 Subjects

When both pregnant women and their partners carry --/αα thalassemia, they have a 25% chance of conceiving an offspring with Hb Bart's hydrops fetalis syndrome. When one of the parents is --/αα, the other is -α/αα or α^T^α/αα or αα^T^/αα, there is a risk of having children with HbH. When parents are both beta-thalassemia carriers, they have a 25% chance of having children with moderate or severe beta-thalassemia. The subjects included in this study are the couples with the above situation.

Here, 467 pregnant Hakka women with thalassemia who sought treatment at the Meizhou People's Hospital (Huangtang Hospital) in Guangdong Province between January 2014 and December 2017. All of them come from 7 areas within Meizhou City, Guangdong Province and their ancestral home is Meizhou. Before the prenatal diagnosis, the genotypes of both father and mother were tested. This study was carried out in accordance with the Declaration of Helsinki and approved by the Ethics Committee of the Meizhou People's Hospital (Huangtang Hospital), Meizhou Hospital Affiliated to Sun Yat-sen University.

### 2.2 Hematological analysis and hemoglobin electrophoresis analysis

Samples were obtained via venipuncture of an antecubital vein using Ethylenediaminetetraacetic acid (EDTA) anticoagulant tube collection, and 2 mL peripheral blood were collected for relative detection. Erytrocyte correlation indices were determined followed the standard operating procedures provided by Sysmex XE-2100 blood analyzer (Sysmex, Inc., Japan). Subjects with low mean corpuscular volume values (MCV) (<82 fl) or Mean corpuscular hemoglobin values (<27 pg) were considered possible thalassemia carriers.

Hemoglobin electrophoresis analysis was detected according to standard laboratory procedures by Sebia capillary electrophoresis system (Sebia, Inc., France). Subjects with low HbA_2_ (<2.5%) were considered possibly α-thalassemia carriers, with high HbA_2_ (>3.5%) were considered possibly β-thalassemia carriers, respectively.

### 2.3 Fetal samples collection and pretreatment

There are 3 main ways to obtain fetal samples: chorionic villus sampling (CVS) was conducted at 10 to 12 weeks of gestation; amniotic fluid (10 mL) was collected at 15 to 22 weeks; cord blood (1.0–2.0 mL) was sampled at 18 to 28 weeks. All procedures were performed under ultrasonography guidance. Meanwhile, 2 ml peripheral blood of the mother was sampled for short tandem repeats (STR) analysis.

Villi were washed twice with normal saline. Villus tissue was removed from 15 mL centrifuge tubes with hook tweezers, placed in Petri dishes with saline. Parts of villi were removed from stripped maternal decidua with hooks at low magnification. Some of the tissues were used for the extraction of fetal DNA. The remaining villi were soaked in saline and frozen at −80°C. Amniocentesis fluid cells were isolated from 10 ml amniocentesis fluid using a centrifuge at 1000 g for 10 minutes. Pipette off supernatant and add 800 ml sterile saline to resuspend the cell pellets. Then take 400 μL of pellet for the extraction of DNA. Take 200 μl umbilical cord blood directly extracted the group DNA, similar to extraction of DNA from peripheral blood.

### 2.4 Short tandem repeats (STR) analysis

Genomic DNAs were extracted from peripheral blood of the couples and villus, amniotic fluid or cord blood of the fetuses using the Tiangen DNA extract kit (Tiangen Biotech CO., LTD., China). Maternal cell contamination (MCC) was tested by STR analysis. Fetal samples are always confused by maternal tissue, so STR analysis was conducted before detecting of the fetal samples.^[19,20]^ The STR analysis with the common markers including D19S433, D5S818, D21S11, D18S51, D6S1043, AMEL, D3S1358, D13S317, D7S820, D16S539, CSF1PO, Penta D, D2S441, vWA, D8S1179, TPOX, Penta E, TH01, D12S391, D2S1338 and FGA (Microread Genetics, Beijing, China) using ABI 3500xl Genetic Analyzer (Applied Biosystems, USA). When all the polymorphic alleles of the mother were absent from fetal sample, the fetal sample was considered free from maternal contamination.

### 2.5 Molecular prenatal diagnosis of α- and β-thalassemia

Gap-polymerase chain reaction (gap-PCR) and flow-through hybridization technology (Hybribio Limited, China) were used to detect the α-thalassemia mutations, including deletion of --^SEA^, -α^3.7^, -α^4.2^ and nondeletion of Hb Constant Spring (α^CS^α) (CD142, TAA→CAA), Hb Quong Sze (α^QS^α)(CD125, CTG→CCG) and Hb Westmead (α^WS^α) (CD122, CAC→CAG). Polymerase chain reaction for detection α-thalassemia mutations was performed according to the following protocol: denaturation at 95°C for 15 minutes, and then 35 cycles of amplification, with 40 seconds at 98°C for denaturation, 1 minute and 10 seconds at 64°C for annealing, and 2 minutes and 30 seconds at 72°C for elongation.

Mutation analysis for the β-globin gene were performed using the polymerase chain reaction (PCR) and flow-through hybridization technology (Hybribio Limited, China), including 16 common nondeletion β-globin gene mutations: CD41-42(-TCTT), CD43(G→T), IVS-II-654(C→T), CD17(A→T), CD14-15(+G), -28(A→G), -29(A→G), CD71-72(+A), βE(G→A), IVS-I-1(G→T), IVS-I-1(G→A), CD27-28(+C), IVS-I-5(G→C), Cap+40-43(-AAAC), initiation condon (T→G) and CD31(-C). Polymerase chain reaction for detection β-thalassemia mutations was performed according to the following protocol: 37 °C for 5 minutes, initial denaturation at 94°C for 4 minutes, and then 40 cycles of amplification, with 30 seconds at 94 °C for denaturation, 30 seconds at 55 °C for annealing, and 30 seconds at 72 °C for elongation. Flow-through hybridization was operated according to the manufacturer's instructions.

### 2.6 Follow-up and statistical analysis

We consulted the clinical records of the subjects when performed molecular prenatal diagnosis. Fetuses with nonsevere thalassemia by genetic diagnosis and were chosen to continue pregnancy were followed up 6 to 8 months after birth. SPSS statistical software version 19.0 was used for data analysis. Data were reported with the descriptive statistics method and showed as the means ± SD.

## 3 Results

Between January 2014 and December 2017, prenatal diagnosis was performed in 467 fetuses of 465 mothers. The age group for fathers and mothers ranged from 18 to 50 years (30.55 ± 5.81) and 16 to 42 years (27.72 ± 5.34), respectively. The mean ages for mothers carried α, β and α compound β globin mutation gene were 27.69 ± 5.19, 27.69 ± 5.83 and 28.13 ± 5.58, respectively. The mean gestational age carrying out the invasive procedure was 16 to 42 weeks (18.47 ± 4.93). Hb A_2_ was showed less than 3.5% or between 2.5% and 3.5% in the parents with α-thalassaemia. The parents with β-thalassaemia showed that Hb A_2_ was beyond 3.5% and mean corpuscular volume (MCV) was <80fl and/or mean corpuscular hemoglobin (MCH) was <27 pg. And the results of 467 pregnant women of hematological screening showed in Table 1.

**Table 1:**
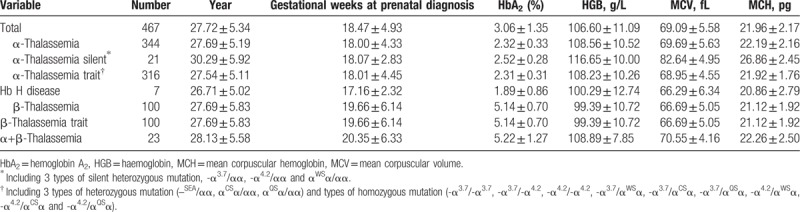
Results of 467 pregnant women of hematological screening.

In the 465 couples, there were 642 subjects (69.03%) that carry Southeast Asian type deletion (--^SEA^), in which included 281 couples. According to previous researches, most of regions indicated a similar feature for higher prevalence genotype of --^SEA^ deletion.^[3,21–24]^ Such couples have 25% chance to produce fetus/fetuses with Hb Bart's. In this study, we identified 69 (19.38%) fetuses with Bart's hydrops syndrome.

Around 467 fetus at-risk pregnancies were performed prenatal diagnosis. We detected 88 CVS samples, 375 amniocentesis fluid samples and 4 cord blood samples (Table 2). The 356 fetuses in α-thalassemia families consisted of 69 (19.38%) with Bart's hydrops syndrome, 20 (5.62%) with Hb H disease, 184 (51.68%) with heterozygote (Table 3). And the 111 fetuses in β-thalassemia families consisted of 31 (27.93%) thalassemia major, 51 (45.95%) with heterozygote (Table 4). There are 13 cases with α+β-thalassemia, including 2 cases with severe β-thalassemia (Table 5).

**Table 2:**
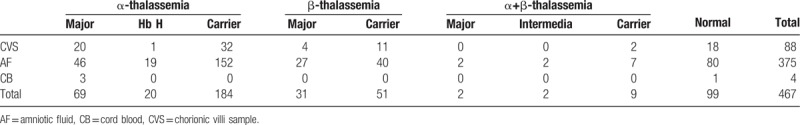
Results of prenatal diagnosis for thalassemia by DNA analysis.

**Table 3:**
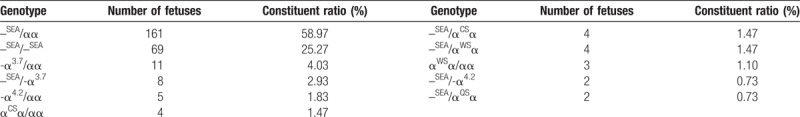
Results of prenatal diagnosis for α-thalassemia.

**Table 4:**
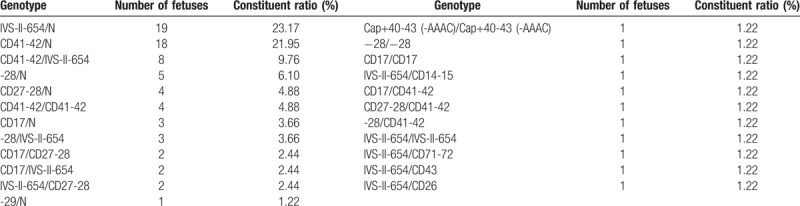
Results of prenatal diagnosis for β-thalassemia.

**Table 5:**
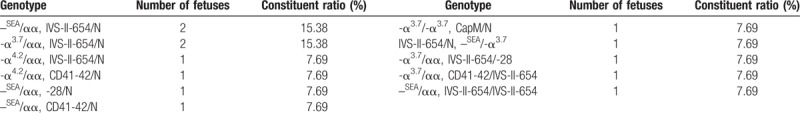
Results of prenatal diagnosis for α+β-thalassemia.

After molecular prenatal diagnosis completed, 69 fetuses were diagnosed as Bart's hydrops syndrome (--^SEA^/--^SEA^), 33 fetuses with severe β-thalassemia and accepted the termination of pregnancy under informed consent. In addition, we detected 20 cases with HbH disease, ten fetuses with deletion HbH disease (8 with --^SEA^/-α^3.7^ and 2 with --^SEA^/-α^4.2^), 4 fetuses with --^SEA^/α ^WS^α, 4 fetuses with --^SEA^/α^CS^α and 2 fetuses with --^SEA^/α^QS^α. The pregnancy of 4 fetuses with --^SEA^/ α^CS^α, 2 fetuses with --^SEA^/α^QS^α and one fetus with fetal malformation (--^SEA^/-α^3.7^ at the same time) were terminated in time. The remaining parents were determined chose to continue pregnancy, and the infants showed no severe anemia phenotype according follow-up within the first half year after birth.

**Figure 1:**
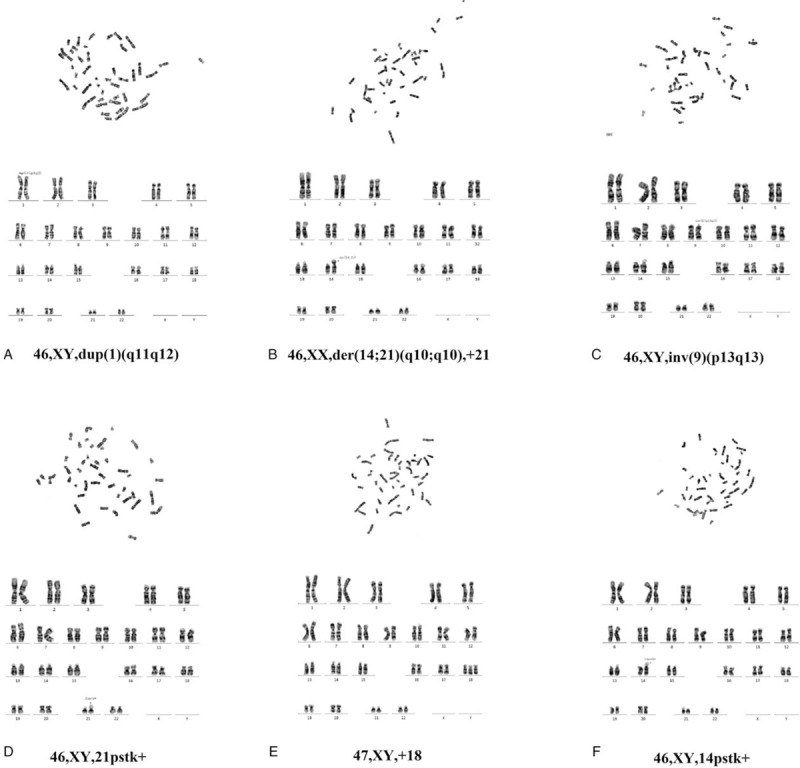
Prenatal karyotype analysis in some fetuses.

According to the results of karyotype analysis of fetal samples, a karyotype of 46,XY,dup(1)(q11q12) (--^SEA^/αα, β^N^/β^N^ at the same time), a karyotype of 46,XX,der(14;21)(q10;q10),+21 (--^SEA^/αα, β^N^/β^N^ at the same time), 2 karyotype of 46,XY,inv(9)(p13q13) (--^SEA^/αα, β^N^/β^N^ and αα/αα, CD27-28/N, respectively), a karyotype of 46,XY,21pstk+ (--^SEA^/αα, β^N^/β^N^ at the same time), a karyotype of 47,XY,+18 (αα/αα, β^N^/β^N^ at the same time) and a karyotype of 46,XY,14pstk+ karyotype (--^SEA^/--^SEA^, β^N^/β^N^ at the same time) we found. The pregnancy of fetuses with 46,XX,der(14;21)(q10;q10),+21; 47,XY,+18 and 46,XY,14pstk+ karyotype (compound --^SEA^/--^SEA^, β^N^/β^N^ thalassemia) were terminated the pregnancy. The results of karyotype analysis showed in Figure 1.

All the mothers with fetal severe thalassemia or other abnormalities chose to terminate pregnancy with informed consent, at which time fetal tissues were examined by molecular diagnosis and the results were confirmed. Fetuses with nonsevere thalassemia by genetic diagnosis and were chosen to continue pregnancy were followed up 6 to 8 months after birth, asked about their growth and development, anemia symptoms and so on. Because most they did not return to the hospital after birth for thalassemia gene testing, have not the comparison between the results of prenatal and post natal genetic tests. However, according to the growth and development and other phenotypes, it is consistent with our prenatal genetic diagnosis, which confirmed the reliability of molecular prenatal diagnosis. The comparison between the results of prenatal and post natal genetic tests is the next step we need to accomplish.

## 4 Discussion

Thalassemia serves as an endemic disease that mainly populars in Guangdong, Guangxi and Hainan province, resulting in numerous health expenditure. According to the epidemiological results, it showed high prevalence of carriers of α thalassaemia (8.53%), β thalassaemia (2.54%), and both α and β thalassaemia (0.26%) in Guangdong province of China. Overall, 11.07% of the population in this area was heterozygous carriers of α and β thalassaemia.^[5]^ Meizhou is located in eastern of Guangdong province in which resident population was up to 5.28 million and annual birth rate reached 12.45‰ (official web site for health and family planning of Meizhou City). Meizhou is a mountainous city, and the vast majority of the permanent residents are Hakka people. Due to geography, culture, customs, and other reasons, the proportion of intermarriage between Hakka people in Meizhou and other regions is relatively small, which consequently triggered higher genetic frequency of thalassemia and that will cause a public health burden in this region. It has been reported that the incidence of thalassemia in Meizhou, Guangdong Province, higher than the average level of the Guangdong Province. The spectrum of both alpha- and beta-thalassaemia mutations is similar to that previously described in southern China, such as Shenzhen, Guangzhou and Hong Kong. Prenatal diagnosis is one of the most effective and direct method to prevent from inherited disease, including thalassemia.

In this study, there are 642 subjects in which 281 couples were detected as heterozygous of the Southeast Asian type deletion (--^SEA^), accounting for 69.03%. Showing that the --^SEA^ deletion is the most common type of thalassemia in this area. Such couples have a 25% chance to give birth to fetus with Hb Bart's syndrome. In this study, we identified 69 fetuses with Hb Bart's hydrops syndrome, accounting for 14.78%, and 33 with severe β-thalassemia (2 with α compound β-thalassemia). And all accepted the termination of pregnancy according to selection principle after informed consent. Especially, we prevented the 33 newborn births with β-thalassemia major syndrome by prenatal diagnosis, which would cost expensive treatment if pregnancies had continued and given birth.

HbH disease can be divided into 2 types according to genotype, deletion HbH disease and nondeletion HbH disease. The vast majority of nondeletion mutations are in the strong function of α2 gene, so the clinical manifestation of nondeletion HbH disease more serious than deletion HbH disease, except --^SEA^/α^WS^α. The clinical phenotype of deletion HbH disease and genotype --^SEA^/α^WS^α variation occurred from mild anemia to transfusion dependent to severe anemia,^[25–27]^ the parents to decide whether to continue pregnancy after prenatal diagnosis. We detected 20 fetuses with HbH disease, ten fetuses with deletion HbH disease, 4 fetuses with --^SEA^/α^WS^α, 4 fetuses with --^SEA^/α^CS^α and 2 fetuses with --^SEA^/α^QS^α. The pregnancy of 4 fetuses with --^SEA^/ α^CS^α, 2 fetuses with --^SEA^/α^QS^α and one fetus with fetal malformation (--^SEA^/-α^3.7^ at the same time) were terminated, the remaining parents continue to pregnancy. They showed no severe anemia phenotype within the first half year after birth and showed good growth according the follow-up visit.

In this study, we identified 2 fetuses with Hb Bart's disease and 1 fetus with deletion Hb H disease, in whom one of his/her parent was α-thalassemia carrier and the other was α+β-thalassemia carrier. These results indicate the existence of at-risk of α-thalassmia homozygous in their fetuses. We suggest that it should be necessary to detect for α-thalassemia mutations in β-thalassemia couple when the other company of the couple is α-thalassemia carrier, for there is 25% chance to produce a fetus with Hb Bart's or Hb H disease.

In addition, 2 pregnant women with fetal abnormalities were detected with ultrasonography. Molecular prenatal genetic diagnosis of thalassemia in these 2 fetuses were αα/αα, β^N^/β^N^ and --^SEA^/αα, β^N^/β^N^, respectively. However, amniotic fluid culture and karyotype analysis showed that the number of fetal chromosomes was 47, XY, +18 and 46, XX, der (14; 21) (q10; q10), +21, respectively. One fetus with nuchal translucency (NT) was thickening, and karyotype analysis showed 46, XX, inv (9) (p13q13). These finally end up with terminate pregnancy after informed consent. These cases suggest that, while prenatal diagnosis and genetic counseling of thalassemia are able to carry out in families, attention also could be paid in the prenatal diagnosis indications of other genetic diseases to avoid missed diagnosis of other genetic diseases.

In this study, to eliminate the risk of maternal cell contamination, we performed DNA analysis twice. The first analysis was done after amniocentesis or CVS, and the second analysis was carried out after fetal DNA was re-extracted from amniocytes or CVS after culture. When we used STR detection technology to rule out maternal tissue contamination, we performed DNA analysis twice after confirmed no maternal cell contamination. With these methods, we successfully minimized the risk of maternal cell contamination to guarantee the accuracy and reliability of results.^[19,28]^ We also retrospectively analyzed the data of prenatal diagnosis of α- and β- thalassemia from 2014 to 2016.^[29]^ Summarizing and analyzing our data are helpful to provide reference and prenatal diagnosis ability for our future work.

## 5 Conclusions

In this study, DNA-based diagnosis was performed on the tissues of fetuses who have risk with severe thalassemia using polymerase chain reaction (PCR) and flow-through hybridization technique. We identified 69 fetuses with Hb Bart's hydrops syndrome and 33 cases with severe β-thalassemia, were terminated the pregnancy. Follow-up visits were performed in 6 to 8 months after the fetuses with nonsevere thalassemia were born. Our findings provide key information for clinical genetic counseling of prenatal diagnosis for thalassemia major in Hakka pregnant women. Our work plays an important role in the prevention and control of thalassemia in Hakka population. We will also try to combine with other techniques to further improve our molecular prenatal diagnostic capabilities, including next-generation sequencing (NGS), Sanger sequencing and MLPA.

## 6 Contributions

Pingsen Zhao conceived and designed the experiments. Heming Wu conducted the laboratory testing. Huaxian Wang, Wei Guo, Zhiyuan Zheng, Huichao Zhu and Jie Wu helped to collect data. Liubing Lan and Mei Zeng recruited subjects, collected clinical data. Pingsen Zhao and Heming Wu prepare the manuscript. Pingsen Zhao reviewed the manuscript.

## Acknowledgments

The author would like to thank other colleagues whom were not listed in the authorship of Clinical Core Laboratory and Center for Precision Medicine, Meizhou People's Hospital (Huangtang Hospital), Meizhou Hospital Affiliated to Sun Yat-sen University for their helpful comments on the manuscript.

## Author contributions

**Conceptualization:** Pingsen Zhao.

**Data curation:** Heming Wu, Pingsen Zhao.

**Formal analysis:** Heming Wu, Huaxian Wang, Zhiyuan Zheng, Pingsen Zhao.

**Funding acquisition:** Pingsen Zhao.

**Investigation:** Heming Wu, Huaxian Wang, Zhiyuan Zheng, Jie Wu, Pingsen Zhao.

**Methodology:** Heming Wu, Huaxian Wang, Liubing Lan, Mei Zeng, Wei Guo, Zhiyuan Zheng, Huichao Zhu, Jie Wu, Pingsen Zhao.

**Project administration:** Pingsen Zhao.

**Resources:** Heming Wu, Huaxian Wang, Liubing Lan, Mei Zeng, Wei Guo, Huichao Zhu, Pingsen Zhao.

**Software:** Heming Wu, Huaxian Wang, Wei Guo, Huichao Zhu, Jie Wu, Pingsen Zhao.

**Supervision:** Pingsen Zhao.

**Validation:** Heming Wu, Huaxian Wang, Pingsen Zhao.

**Visualization:** Heming Wu, Pingsen Zhao.

**Writing – original draft:** Heming Wu, Pingsen Zhao.

**Writing – review & editing:** Pingsen Zhao.
